# The role of awareness in shaping responses in human visual cortex

**DOI:** 10.1098/rsos.230380

**Published:** 2023-08-09

**Authors:** Zien Huang, Poutasi W. B. Urale, Catherine A. Morgan, Geraint Rees, D. Samuel Schwarzkopf

**Affiliations:** ^1^ School of Optometry and Vision Science, University of Auckland, Auckland, New Zealand; ^2^ School of Psychology and Centre for Brain Research, University of Auckland, Auckland, New Zealand; ^3^ Centre for Advance Magnetic Resonance Imaging, Auckland UniServices Limited, Auckland, New Zealand; ^4^ UCL Institute of Cognitive Neuroscience, University College London, London, UK; ^5^ Experimental Psychology, University College London, London, UK

**Keywords:** awareness, unconscious processing, perceptual grouping, functional magnetic resonance imaging, object recognition, spatial integration

## Abstract

The visual cortex contains information about stimuli even when they are not consciously perceived. However, it remains unknown whether the visual system integrates local features into global objects without awareness. Here, we tested this by measuring brain activity in human observers viewing fragmented shapes that were either visible or rendered invisible by fast counterphase flicker. We then projected measured neural responses to these stimuli back into visual space. Visible stimuli caused robust responses reflecting the positions of their component fragments. Their neural representations also strongly resembled one another regardless of local features. By contrast, representations of invisible stimuli differed from one another and, crucially, also from visible stimuli. Our results demonstrate that even the early visual cortex encodes unconscious visual information differently from conscious information, presumably by only encoding local features. This could explain previous conflicting behavioural findings on unconscious visual processing.

## 1. Introduction

Even when participants are unaware of a visual stimulus, a considerable amount of information about it is preserved in the brain. Contextual modulations of orientation, object size and brightness occur even when masking stimuli inducing these illusions [1–6], suggesting contextual processing does not require awareness. After-effects evoked by rapidly alternating images demonstrate that cortical mechanisms track colour faster than perception [7]. Brain imaging has also shown that even when a stimulus is rendered invisible by masking, the primary visual cortex (V1) still encodes its orientation [3]. Nonetheless, the nature of object representations in the absence of awareness remains unclear. Ample evidence shows that some processing of visual objects occurs unconsciously [8]. Invisible stimuli activate higher object-selective brain areas [9–11] but responses are more variable [12,13] than during conscious perception. Activity patterns in ventral visual cortex measured while participants view complex images differ between visible and invisible stimuli [10]. Precise psychophysical measurements also demonstrated reduced orientation selectivity for gratings concealed from awareness during binocular rivalry [14].

Unconscious visual processing could differ from conscious perception in the extent to which it integrates local visual features into a global representation. There is evidence that Kanizsa shapes defined by illusory contours, indicative of a perceptual organization process inferring a shape from discontinuous features, are processed unconsciously under conditions of masking [15,16] and during the attentional blink [17,18]. However, in previous work, we found that discriminating illusory contours, and therefore presumably the perception of such contours, requires awareness of the inducing stimuli [1,19].

**Figure 1.**
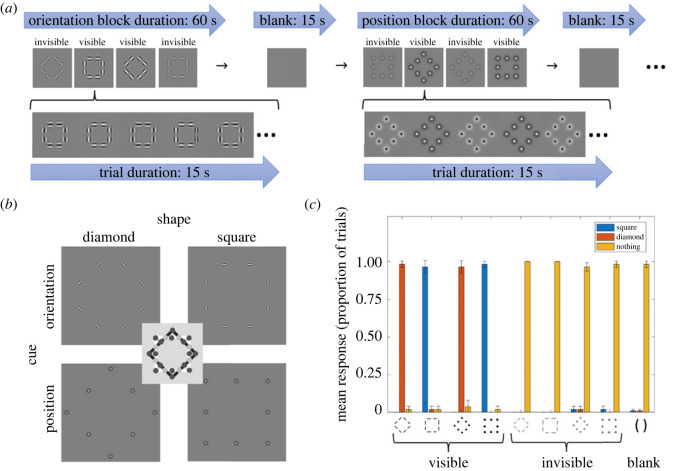
Experimental design and behavioural results. (*a*) Typical trial sequence. Position and orientation cue stimuli were shown in blocks, lasting 60 s in total (top row). Each block comprised four trials lasting 15 s each. In any given stimulus trial, a shape counter-phase flickered for the whole duration (the second row shows zoomed-in example sequences for an orientation-defined square and a position-defined diamond, respectively), with the flicker rate determining its visibility. All four combinations of shape (diamond versus square) and visibility (visible versus invisible) for a given cue appeared within a given block in pseudo-random order. These 60 s stimulus blocks were interleaved with 15 s blank trials. Blocks for the two cues (orientation versus position) always alternated across an experimental run. (*b*) Orientation versus position stimuli. Shape stimuli could be formed either by orientation or position cues. The shape is either a square or a diamond (columns of images). Only the orientation of the elements provides shape information at the intersections of the two shapes (top row of images, as also denoted by light grey circles in the central schematic). By contrast, at the corners and in the middle of the sides (dark grey circles in central schematic), orientation is at best ambiguous and shape discrimination requires position information (see bottom row of images) [20]. (*c*) Behavioural mean response rates across participants for each stimulus type. Bars plot the proportion of trials that participants gave the three possible responses (see colour scheme). Error bars indicate ±1 s.e. of the mean across all analysed participants. As expected, participants performed accurately for visible conditions, and correctly identified that nothing was presented on blank trials. However, they consistently reported seeing nothing during invisible trials.

We previously investigated specifically whether stimulus awareness influences the integration of local features into a global shape representation [20]. In this earlier work, participants were primed with a shape stimulus and then performed a shape-discrimination task on a subsequent probe shape; specifically, they reported whether the probe was a diamond or a square. The stimulus design allowed us to disentangle the effects of local stimulus features compared with the representation of the global shape. As figure 1*b* shows, shapes were defined either by the position of elements without orientation information (bottom row of images), or by the orientation of local elements without any position information (top row of images). The central schematic in figure 1*b* explains the locations of the elements in the two shapes. Dark grey dots correspond to position cues, which differ between the square and diamond shapes. By contrast, light grey dots correspond to the orientation cues that are located on the intersection of the square and diamond shape. Here, the position is uninformative; it is only possible to tell apart the shapes by relying on the edge orientation.

Visible primes showed a substantial priming advantage, with faster response times when the probe and prime had the same shape, regardless of the defining cue. However, rendering the prime invisible via fast counterphase flicker [4] revealed a dissociation: position-defined probes benefited only from position-defined primes. Surprisingly, orientation-defined primes only primed performance to position-defined probes. This cross-cue priming effect was confined to retinotopic coordinates. Invisible orientation primes without a global shape interpretation also did not improve performance for discriminating visible orientation probes. Taken together, these results suggested three things. First, awareness is necessary to process global shape. Second, the brain represents retinotopic stimulus position in the absence of awareness. Third, orientation-defined shape might also recruit retinotopic circuits (such as intrinsic lateral connections) in visual cortex but without any global shape representation.

Here, we tested these three predictions with functional magnetic resonance imaging (fMRI). We hypothesized that the signature of retinotopic activity in visual brain regions should reflect the stimulus shape (square or diamond). We used a retinotopic atlas to project responses evoked by these stimuli back into visual space (see [21–24] for examples). Moreover, we compared the representational similarity of response patterns within individual brain regions to test if they encoded global shape information.

## 2. Material and methods

### 2.1. Participants

Eight participants (age range: 22–44 years, four female, one left-handed) with normal or corrected-to-normal visual acuity participated in the study. All gave written informed consent. The University of Auckland Human Participants Ethics Committee approved experimental procedures. One participant (008) was excluded from fMRI analysis because their behavioural results indicated the stimulus manipulation was ineffective for them (see Results).

### 2.2. Apparatus

We acquired brain images using a MAGNETOM Vida Fit 3 Tesla magnetic resonance imaging (MRI) scanner (Siemens Healthcare, Erlangen, Germany) at the Centre of Advanced Magnetic Resonance Imaging (CAMRI) at the University of Auckland. To record key presses, we used an MRI-compatible button box. Stimuli were displayed on a gamma-corrected 32-inch wide-screen liquid crystal display (BOLD Screen, Cambridge Research Systems Ltd.) at a resolution of 1920 × 1080 pixels at the back of the scanner bore and viewed through a head-mounted mirror (approximate viewing distance: 114.5 cm). The screen thus subtended 34.5° × 19.0° of visual angle.

### 2.3. Stimuli

Stimuli were generated and displayed using Matlab (MathWorks; version R2017a, 9.2.0.538062) and Psychtoolbox (version 3.0.14). Orientation and position stimuli consisted of eight local elements arranged in either a square or a diamond (a square rotated by 45°). As in a previous study [20], our stimulus design exploited unique locations on the shape (figure 1*b*). The position of the elements at the intersections of the two shapes (denoted by light grey circles in the central schematic) are identical for squares and diamonds; therefore, elements must contain orientation cues to enable shape discrimination. By contrast, the position of the elements at the corners and the middle of each side of the squares (dark grey circles in the central schematic) are the most informative while orientation cues are at best ambiguous. Thus, shape stimuli could be defined either by orientation (top row of images in figure 1*b*) or position cues (bottom row), and our stimulus design enabled us to isolate the impact of local stimulus features from the global form representation. In addition, a blank stimulus contained only a uniform grey screen.

Throughout each 15 s trial, the stimuli were presented continuously alternating between positive and negative contrast polarity at different temporal frequencies (visible trials: approx. 3.33 Hz; invisible trials: 120 Hz). Therefore, during invisible trials participants should only perceive a grey screen, but during visible trials they saw flickering stimuli [4]. The side length of the square/diamond shapes was 5.3°. Orientation-defined shapes elements were Gabor patches (s.d. = 0.09°, wavelength = 0.18°). Position-defined shapes were two-dimensional difference-of-Gaussian (sombrero) functions (s.d._1_ = 0.09°, s.d._2_ = 0.04°). The contrast of the elements was 20%.

### 2.4. Procedure

The experiment consisted of four runs. Each run included four blocks (two each for orientation and position). Orientation blocks and position blocks alternated across the duration of the run, separated by blank trials (figure 1*a*, top row). Therefore, there were in total eight trials for each stimulus condition across the whole experiment, and 16 blank trials. Odd-numbered runs started with orientation blocks; even-numbered runs started with position blocks. Each block comprised four trials, which were always one of the four conditions (diamond versus square, visible versus invisible), and the order of the trials within each block was pseudo-randomized. The expanded sequences in second row of figure 1*a* show examples of trials in the orientation-defined square or position-defined diamond condition, respectively.

Participants indicated via the button box whether they saw a square, diamond, or nothing. They were instructed to respond as accurately as possible rather than respond as quickly as possible. Participants only needed to press a button when they saw the image change, so whenever the trial changed. However, they were free to press the button more often (for example, when they forgot if they had already pressed it or if they briefly perceived something). If they did not press any button after a new trial started, we counted this trial as having the same response as the previous trial. This accounted for the fact that they might not see anything in different trial types: for example, if an invisible shape trial followed a blank trial, they would have already indicated in the previous trial that they saw nothing. Throughout the run, participants were asked to fixate on a dot (diameter: 0.1°) in the centre of the screen.

### 2.5. Magnetic resonance imaging acquisition

All functional images were acquired with a 32-channel head coil. For each of the four experimental runs, 300 T2*-weighted image volumes were acquired (TR = 1000 ms, TE = 30 ms, flip angle = 62°, a multi-band/slice acceleration factor of 3, an in-plane/parallel imaging acceleration factor of 2, rBW = 1680 Hz/pixel, 36 slices, matrix size = 96 × 96 voxels) with 2.3 mm isotropic voxels. Slices were centred on, and oriented approximately parallel to the calcarine sulcus. Dummy volumes prior to each run to allow the signal to reach equilibrium were acquired by default, but not saved. For each participant, we also collected an anatomical scan (a T1-weighted anatomical magnetization-prepared rapid acquisition with gradient echo scan, with a 1 mm isotropic voxel size and full brain coverage).

### 2.6. Data preprocessing

We processed all functional data in SPM12 (Wellcome Centre for Human NeuroImaging), using default parameters to perform mean bias intensity correction, realignment-and-unwarping of motion-induced distortions and coregistration to the structural scan. Using FreeSurfer (version 7.1.1), we automatically segmented and recreated the pial and grey-white matter borders as three-dimensional surface meshes. We then inflated these mesh surfaces into a smooth and a spherical model [25].

### 2.7. General linear model estimation

We recorded blood oxygenation level dependent (BOLD) responses across the brain, except for some parietal regions that were typically outside the imaged region. Separately for each participant, the concatenated time series of the fMRI experiments were entered into a voxel-wise general linear model using SPM12. Boxcar regressors were defined per condition and run and convolved with the canonical haemodynamic response function. We also included six regressors of no interest for the motion correction parameters from realignment, plus a separate global regressor for each run. After model estimation, the neural activity for orientation and position as well as visible and invisible stimuli was separately contrasted against the (implicit) baseline. We also calculated contrasts for these stimulus conditions separately for odd- and even-numbered runs. Finally, we projected these contrast images from volume space to the surface mesh by locating the voxel halfway between each vertex of the pial and grey-white matter surfaces.

### 2.8. Retinotopic back-projection analysis

We also generated a prediction of retinotopic maps for visual regions V1, V2 and V3, using a probabilistic atlas procedure [26], based on the finding that an anatomical image alone can predict the retinotopic arrangement of early visual cortex for an individual with the same level of accuracy as 10–25 min of functional mapping. Next, we examined whether visual cortex represents global shapes in the absence of awareness. We projected the brain activity evoked by each stimulus type back into visual space [21,24], using a procedure implemented in our SamSrf 9 toolbox (https://osf.io/2rgsm). Briefly, this entails moving a circular searchlight (radius: 1°) through the visual field in steps of 0.05°, averaging the contrast values for the population receptive fields (pRFs) whose centre fell inside the searchlight, and plotting the mean activity for each searchlight position. We did this separately for regions V1, V2 and V3, pooling the data across all participants.

### 2.9. Representational similarity analysis

Moreover, we assessed the similarity of response patterns in different brain regions. Specifically, we used regions V1, V2 and V3 from the probabilistic retinotopic atlas. In addition, we also used an atlas based on group-average maps [27] warped back into native brain space for each participant to automatically delineate larger clusters for V4, V3A/B and the lateral occipital areas (LO). In each region of interest, we then calculated the correlation matrix of vertex-wise response patterns for all stimulus conditions comparing the responses in odd- and even-numbered runs, respectively. In the retinotopically defined regions V1–V3, we restricted this representational similarity analysis to vertices with pRFs whose eccentricity was between 1 and 6°, to broadly cover the locations of the stimuli while excluding random background responses. For the higher regions V4, V3AB and LO, where we had no retinotopic maps, we instead restricted this analysis to vertices showing the top 5% response across all stimulus conditions.

Correlation matrices were computed separately in each participant. We then applied Fisher's *z*-standardization to linearize the correlation coefficients and folded over the matrix into the upper-right triangle by averaging the correlations for equivalent cells (i.e. the odd-versus-even and even-versus-odd correlation for any given comparison). Finally, for visualizing these representation similarity matrices at the group level we averaged correlations across participants. With a sample of seven participants, conventional group-level statistics would only have statistical power to detect relatively large effects. However, such a statistical approach is generally ill-suited for research like this because it ignores the high reliability of results at the individual level. Our aim here was to reveal effects that are consistently detected in most individuals, thus effectively treating each participant as a replication. To this end, we first established the significance level of each correlation test *within each participant*, using an *α* = 0.001, Bonferroni-corrected by the number of comparisons in each region of interest (i.e. 36 or 10 comparisons, depending on the analysis). As evidence that a given cell supported the presence of a correlation, we then used a highly conservative criterion that at least five of the seven participants must show a significant effect with the same sign as the group average.

### 2.10. Sensitivity analysis

To estimate the minimal effect sizes our small sample approach could detect, we conducted a sensitivity analysis. We determined the number of data points (vertices) across all participants and regions of interest that gave rise to the correlations for each individual participant. In the early retinotopic cortex (V1–V3), we selected vertices from the retinotopic atlas whose pRFs overlapped with our stimuli, resulting in 2927–5493 (mean ± s.d.: 4004 ± 813) vertices. In the higher extrastriate regions (V4, V3A/B and LO), we selected only the top visually responsive vertices and the numbers were an order of magnitude smaller, resulting in 105–304 (180 ± 53) vertices. We therefore estimated sensitivity separately for these two sets of brain regions.

The minimum number of vertices gives a lower bound for the statistical power achievable within individual participants. The power of our analysis approach is then given by the binomial probability of reaching the criterion level of significant correlations in five out of seven participants for any given possible correlation between 0 and 1. The small-sample approach effectively boosts sensitivity for robust effects where power is high in individual participants, but it is highly conservative for unreliable effects that are not consistently detected in individual participants. This analysis (electronic supplementary material, figure S1) reveals that in the early visual cortex we had 80% power to detect weak true effect sizes of *r* ∼ 0.1. By contrast, in the higher regions we only 80% power to detect moderate effect sizes or *r* ∼ 0.45.

## 3. Results

### 3.1. Behavioural performance on shape discrimination task

Participants performed a simple shape discrimination task in the scanner. We anticipated that during visible trials, participants' accuracy would be at ceiling. By contrast, during invisible trials, participants should see nothing and be unable to distinguish between shapes. Four participants performed the task perfectly; that is, they correctly identified visible shapes in each of the eight trials, and always reported seeing nothing for all eight invisible trials and all blank trials. The exceptions were participant 001, who correctly reported seeing the position-defined square and diamond in one invisible trial each, respectively. Participant 005 incorrectly reported seeing a diamond in one trial when an invisible position-defined square was presented. Participant 004 never reported seeing any shape during invisible or blank trials but occasionally misidentified visible shape stimuli or reported seeing nothing. Given the simplicity of the task, this could have been due to momentary fluctuations in arousal. However, their performance on all visible trials was still high, achieving at least six of eight trials correctly across all conditions.

Only participant 008 performed poorly across all stimulus conditions. Specifically, the proportion of correct responses for invisible trials should be close to zero, well below the chance level of 33.3%. If a participant did not meet these criteria, we can infer that they could see the shapes during the invisible trials, that they could not see the shapes during the visible trials, or that were simply not following task instructions. We therefore excluded participant 008, as their response accuracy for invisible trials (detecting the shape presented on a given trial) was greater than 33.3%; this could have been due to unstable fixation rendering the masking ineffective. Their response accuracy for the visible orientation-defined stimuli was also lower than 66.6%. Many participants reported during debriefing that the visible orientation stimuli appeared very faint and may have produced some Troxler fading despite the slow counterphase flicker. However, this evidently did not prevent other participants from discriminating the visible shapes correctly. Figure 1*c* shows the mean response rates of all participants (excluding 008) to each stimulus type.

### 3.2. Different neural signatures for visible and invisible stimuli

We evaluated whether the brain groups position and orientation cues into a signature of global shape in the visual cortex. While we anticipated that the retinotopic response to visible orientation and position cues would be distinct due to the different positions of the cues, we reasoned that the responses to the shape might also partly match the shape itself, either due to feedback or contextual modulations within each region. We projected the brain activity evoked by each stimulus type back into visual space [21,24], using the probabilistic retinotopic maps for regions V1, V2 and V3 [26]. We pooled data across all participants rather than averaging individual back-projections. Individual back-projections inevitably contain gaps due to inaccuracies in sampling functional data to the cortical surface, partial volume artefacts near visual field meridians and vascular artefacts eclipsing patches of the retinotopic map [28].

**Figure 2.**
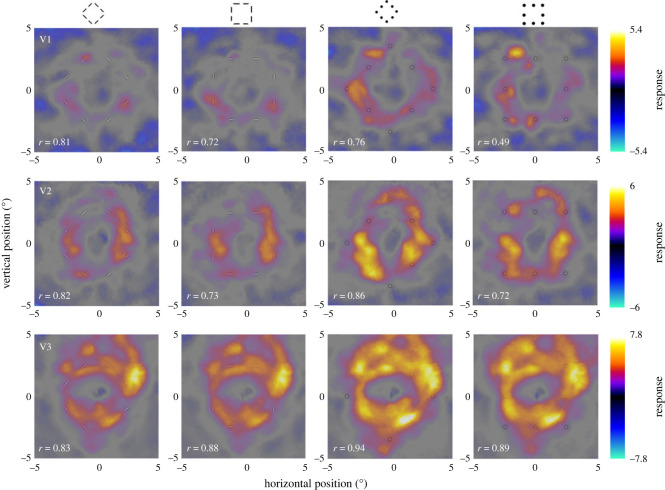
Neural signature of visible stimuli in visual cortex. Brain activity measured in regions V1–V3 with each stimulus condition projected back into visual space. The colour represents the strength of neural responses in each location. The *x*- and *y*-axes represent horizontal and vertical visual field positions, respectively. The icons at the top denote the stimulus condition and are also overlaid in each figure at the same position and size presented. Note that the colour scale varies between regions of interest. The aim of these plots is to visualize the spatial pattern of responses. The absolute amplitude of the response is secondary because the overall response to visual stimuli varies between brain regions.

On visible trials, we expected that retinotopic response signatures resemble the grouped shape of the actual stimuli. However, while there were some noticeable differences between the shapes of neural signatures for position-defined diamond and square in V1 and V2, for orientation-defined stimuli the signatures were very similar (figure 2). The shapes of the neural responses in V3 were generally similar across all four conditions (position versus orientation, diamond versus square). We also quantified the reliability of these back-projections by calculating them separately for odd- and even-numbered runs and then computing the correlation between the two back-projections and extrapolating the test–retest reliability [29]. This revealed robust response signatures (all *r* > 0.49).

**Figure 3.**
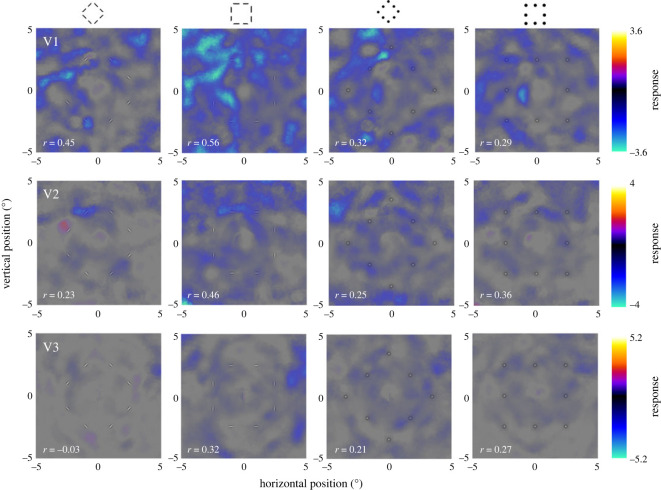
Neural signature of invisible stimuli in visual cortex. All conventions as in figure 2 except for the different scale of the colour scheme because responses to invisible stimuli were considerably weaker than to visible stimuli. As in figure 2, the purpose of these plots is to reveal any spatial patterns of responses, rather than the absolute response level, which differs between brain regions.

Importantly, in invisible trials, neural responses were weak and consistently negative across all three regions (figure 3). Despite that, split-half correlations showed these response signatures were reliable, albeit less so than for visible trials (all 0.21 < *r* < 0.56, except for the invisible orientation-defined diamond in V3 where *r* = −0.03). There was no obvious signature of the grouped shapes although it was possible to make out a faint trace of the (negative) neural response at the location of the position-defined stimuli.

### 3.3. Only visible stimuli share neural representations

**Figure 4.**
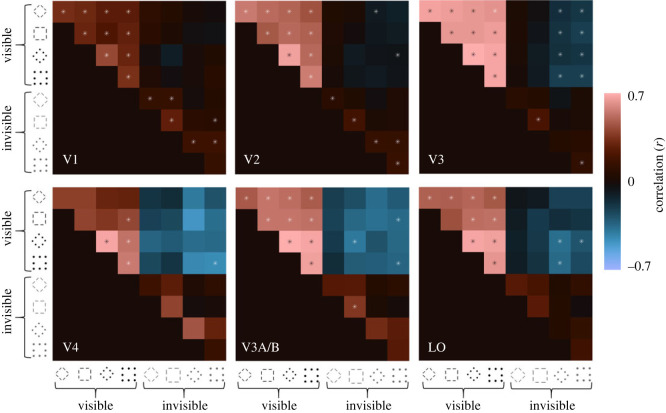
Similarity of neural representation of different stimulus conditions for each brain area. The colour bar at the right-hand side of the image denotes the split-half Pearson correlation coefficient averaged across participants between the neural response patterns for different stimulus types (see icons). Asterisks in each cell indicate correlations that were significant at *p* < 0.001 (Bonferroni corrected) in at least five of the seven individual participants.

Next, we sought to quantify the similarity of response patterns in different brain areas. We conducted a representational similarity analysis for each region of interest by computing the correlation matrix of vertex response patterns in each region for each condition, comparing the responses from odd- and even-numbered runs. We anticipated that the response patterns for all four visible stimulus types (diamond versus square, orientation versus position) were strongly correlated, because their back-projected signatures resembled one another. This was indeed what we found: response patterns for all four visible stimulus types showed strong correlations. Results surpassed our statistical criterion (at least five of seven participants showing Bonferroni-corrected significant correlations at *p* < 0.001) for every comparison across V1–V3 and V3A/B, most comparisons in LO, and primarily for position-defined shapes in V4 (figure 4).

For invisible stimuli, response patterns were consistently correlated between odd- and even-numbered runs within a given condition in most comparisons (diagonal cells in matrix) in V1 and V2. Interestingly, in V1 and for position-defined stimuli in V2, responses patterns for square and diamond shapes were also correlated, albeit relatively weakly. However, there was no compelling evidence of representational similarity cross-cue, between invisible orientation- and position-defined stimuli. In areas V3 and beyond, few correlations between invisible stimulus representations reached the statistical criterion. Note, however, that our sensitivity analysis (electronic supplementary material, figure S1) showed that the analysis of higher extrastriate regions (V4, V3A/B and LO) could only reliably detect moderate correlations (*r* ∼ 0.45). Analysis of these regions therefore may have lacked statistical power to reveal the similarity of response patterns to invisible stimuli. Numerically, the pattern of results for invisible stimuli was similar to the early regions where we had considerable statistical power. Taken together, consistent with the results for back-projections, invisible stimuli produced reliable responses in early visual areas, but unlike for visible stimuli these signatures were largely specific to the local visual feature.

Interestingly, response patterns evoked by invisible stimuli in higher extrastriate regions (V4, V3A/B and LO) tended to be inversely correlated with responses to all visible conditions although only few of these comparisons reached the statistical criterion (although again note the lack of statistical power for the higher extrastriate regions). In V3, we found consistent negative correlations but only for invisible position-defined shapes. A hint of this effect was also evident for some comparisons in V2, although these negative correlations were very weak on average.

### 3.4. Potential orientation-defined shape selectivity for invisible stimuli

Both the back-projections and representational similarity analysis of responses suggested strong and relatively widespread responses to visible stimuli in the earlier regions. Responses to diamond and square stimuli were very similar, regardless of the defining visual feature (orientation versus position). Thus, we observed no evidence of selectivity to the stimulus shape. It is possible that the strong stimulus-evoked responses drowned out subtle signals related to shape processing. These strong signals might also reflect differences in the mental state, such as attentional engagement, between visible and invisible stimuli. We previously suggested the stimuli we used could recruit lateral horizontal connections within retinotopic areas like V1 [20]. However, such signals are probably much weaker than the direct stimulus-driven response. We therefore reasoned such subtle signatures necessitate computing the contrast *between* shapes. We explored this possibility by subtracting response pattern to diamonds from those to squares, separately for each defining cue and for visible and invisible conditions. Then, we conducted another representational similarity analysis but now on these contrast patterns.

**Figure 5.**
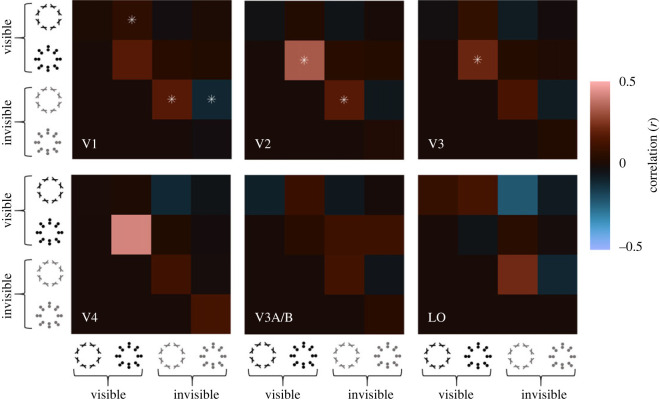
Similarity of neural shape selectivity for each brain area. Correlations calculated after subtracting the response patterns to squares from the response to diamonds. Icons denote the defining visual feature. All other conventions as in figure 4.

Results indicated that these contrasts were reliable between odd- and even-numbered runs in V1–V3 for a few comparisons only (figure 5). Specifically, in V1, the pattern was similar for invisible orientation shapes. There was also a suggestion of similar response patterns between visible orientation- and position-defined shapes. Interestingly, patterns were inversely correlated between invisible orientation and position stimuli. In V2, both visible position-defined shapes and invisible orientation-defined shapes showed consistent response patterns, but no other conditions did. In V3, only visible position-defined shapes reached this criterion. In V4, V3AB and LO no correlation was consistently significant in at least five participants.

## 4. Discussion

We compared neural signatures of perceptual grouping stimuli in visual cortex between conscious and unconscious processing. We measured fMRI responses while participants discriminated the shapes of fragmented diamond and squares defined either by the position or the orientation of their component fragments (figure 1*b*). By projecting these responses back into visual space, we could study the retinotopic stimulus signature. Visible stimuli whose contrast polarity reversed at approximately 3.33 Hz caused robust responses throughout visual cortex. Moreover, the spatial pattern of responses to the different stimulus conditions strongly resembled one another. While the response in V1 and V2 to position-defined stimuli at least vaguely matched the square and diamond shapes presented, orientation-defined stimuli evoked similar responses for either shape, consistent with the absence of distinguishing position information. Thus, we found little evidence of selectivity for stimulus shape in these stimulus-evoked responses. This finding may seem surprising, especially for intermediate and higher extrastriate regions that process complex object information [9]. Visible and invisible stimuli probably differ in terms of the mental state, such as attentional engagement. More importantly, (population) receptive fields are larger as we progress from earlier to higher visual regions [30,31]. This will inevitably blur the retinotopic signature; as we have shown previously [24], even with detailed retinotopic mapping data such neural signatures are very coarse in higher regions like LO. Here, our stimuli comprised tiny, low-contrast fragments. It is likely that these only produced subtle differences between the shapes. Our analysis of the contrast patterns comparing square and diamond stimuli aimed to correct for general activation differences. This indeed revealed shape selectivity for visible position-defined stimuli, at least in V2 and V3.

By contrast, neural responses to invisible stimuli were statistically reliable but weak and were generally deactivations relative to baseline. This could be due to response suppression related to preceding visible stimuli [32]. Such deactivations to invisible or masked stimuli are commonly found, e.g. in a previous study investigating the representation of complex images in the ventral stream [10]. While some retinotopic response signatures to position stimuli might still have contained a faint trace of the stimulus shape, response patterns were very dissimilar between stimulus conditions, especially between orientation- and position-defined stimuli. This suggests they only encoded the local visual features. Crucially, they were also generally dissimilar to representations of visible stimuli; in fact, in V3, and in some comparisons in higher extrastriate cortex, response patterns were even inversely correlated with those for visible stimuli, at least for position-defined invisible shapes. This could be related to the negative responses to invisible stimuli: since visible stimuli evoked response patterns similar to one another, this results in overall negative correlations with the invisible response patterns. It is important to note that these negative correlations could not simply arise due to the overall difference in responses between visible and invisible stimuli. If the response patterns to invisible stimuli were simply the result of noise, they would not be reliable and should not be (anti-)correlated with responses to visible stimuli.

Our findings are broadly consistent with our previous psychophysical priming experiments [20], which suggested that the neural representation of invisible shape stimuli was restricted to simple information encoded in retinotopic coordinates. However, this previous work also suggested that invisible orientation stimuli might recruit local circuits corresponding to the stimulus shape, such as lateral horizontal connections in V1. We therefore hypothesized that there might be a faint trace matching the stimulus shape in retinotopic brain activity for invisible orientation-defined stimuli; however, in the present work, we found no evidence of such traces in the back-projected responses. Our statistical approach of testing for reliable effects in each individual participant was designed to reveal clear representations of our shape stimuli. This analysis was far more sensitive than classical group-level statistics because it takes the reliability of each individual's results into account. However, detecting extremely subtle signals might require testing for weak group averages that can only be demonstrated with group-level statistics and much larger sample sizes. We deem this unlikely, however, because responses to invisible stimuli were reliable and generally negative (figure 3). If the absence of activations along the shape outline were trivially due to insufficient statistical power, we would expect responses to vary randomly about zero and have poor split-half reliability. Indeed, comparing response patterns indicated a degree of shape selectivity to invisible orientation stimuli in early regions. It is possible that this subserves behavioural priming by such stimuli. Lateral connections might mediate this priming effect without causing spikes in neurons whose receptive fields are located along the shape's edges. Rather, they could act to modulate the excitability of these neurons, thus enhancing the response to subsequent probe stimuli. Future neuroimaging research could investigate this by adapting our earlier priming paradigm for use inside the scanner.

Our results also tie in with brain imaging studies showing distinct neural representations during conscious and unconscious processing. V1 encodes the orientation of masked stimuli similar to visible stimuli [3]. However, while higher ventral brain regions also contain representations of invisible complex visual objects [9], fine-grained activity patterns within those regions differ between visible and invisible stimuli [10]. While some studies suggested higher visual cortex shares some representation between visible and invisible stimuli [10,11], unlike our experiments these studies used intact, complex images that did not explicitly require perceptual integration. These experiments therefore cannot rule out that neurons in higher category-selective regions merely respond to the presence of local features related to their preferred category. On that note, the study by Sterzer *et al*. [10] found similar patterns only for a large region of interest combining the fusiform face area and parahippocampal place area. This could be due to subtle univariate response differences between these clusters. Similarly, while invisible images of fearful faces elicit brain responses, demonstrating that unconscious processing conveys not only visual information but also complex emotional content [33], this could also be mediated by coarse, low-level signals [34,35]. Left fusiform gyrus and right parahippocampal gyrus play a role in the unconscious processing of surprised facial expressions that might be mediated by a pathway through the right amygdala and thalamus [36]. In general, cortical activation levels are lower with invisible stimulation compared with visible stimulation [9,37,38], a finding we also replicated here. Previous research also suggested that response patterns to invisible stimuli are more variable [12,13]. Our findings now suggest that this could relate to what these responses encode: while visible shapes activate neurons tuned to complex objects in higher regions, if invisible stimuli instead only drive responses to local visual features, this fragmented representation might also be more variable.

According to this interpretation, when local elements are processed without awareness, neurons respond to individual elements, but only relatively coherent images are processed as global objects. Without awareness, no integration of visual information into shape representations occurs. This, however, disagrees with previous studies that suggested even the meaning of complex scenes can be analysed without conscious awareness. Scenes containing incongruent object relations (such as a basketball player dunking a melon) were faster to break masking in a continuous flash suppression [39] paradigm [40] or impaired response times of subsequently presented target scenes [41]. These findings have, however, been called into question [42,43], and brain activity does not differentiate between masked congruent and incongruent scenes [44]. Similarly, illusory Kanizsa contours, an indication of perceptual integration of fragmented stimuli, have been reported to break through continuous flash suppression faster than control stimuli [15]. While Moors *et al*. [45] replicated this finding, they suggested that no figure-ground assignment is formed during interocular suppression. A lack of feedback loops during unconscious processing could explain why humans cannot recognize shapes without awareness. Feedback transmission from object-selective areas to early visual cortex is essential for perceiving illusory contours [46]. These findings cast doubt on whether the breakthrough time in continuous flash suppression is appropriate for probing unconscious processing [43,47,48]. Unconscious Kanizsa shapes can also produce priming effects [16] similar to our own priming work [20]. Other research has suggested that there is a neural signature of Kanizsa shapes shown during the attentional blink [17,18]. However, these studies cannot distinguish the actual percept of illusory contours from the prerequisite neuronal processing necessary to produce such a percept. While the attentional blink experiments used elegantly designed control stimuli to mitigate this problem, the possibility remains that their results reflect differences in the stimulus configuration. The only way to rule this out is by quantifying whether participants in fact perceived illusory contours [1,19,46].

We note, however, that the attentional blink studies could disentangle awareness from stimulus processing. They relied on spontaneous fluctuations in awareness to test unconscious processing. A general limitation with all investigations using masking to render stimuli invisible, like our present study, is that the masking technique confounds manipulations of the stimulus with awareness. Brightly flashing or dynamic masks must produce strong neuronal responses that might drown out subtle signals related to target stimuli. Exposure to high-contrast visual patterns also causes perceptual after-effects [49]. Positive after-images following a quick flash of light can be highly striking [50]. Masking could therefore affect the processing of concurrently or subsequently presented stimuli. The counterphase flicker method we used here may reduce the signal as it is above the flicker fusion threshold [51]. Our findings nevertheless indicate that counterphase flicker is an effective technique for investigating awareness. We measured reliable fMRI response patterns even during masking and our earlier work showed such stimuli still cause behavioural effects [20]. Even at 160 frames per second, cortical processing of the stimuli still occurs [4]. Nonetheless, future studies could use metacontrast masking by creating a closer phenomenological match between visible and invisible conditions [3,52] and thus reduce any confounding influence of the stimulus manipulation. A better alternative could be to use spontaneous fluctuations in awareness like the attentional blink [17,18]. However, this also reduces the sensitivity of the experiment because it would involve only analysing a small number of brief events. Either way, both these methods would entail a considerable redesign of our experiment.

Another limitation with the present experiments is that our set-up precluded the use of eye-tracking in the scanner. Unstable fixation or blinks could result in failure of the fast-flicker masking. This may indeed explain the results for participant 008 who we excluded because they reported seeing stimuli on several invisible trials (and they also failed to correctly recognize many visual trials). However, eye movements would also break the correspondence between retinotopic cortex and visual field locations, resulting in ill-defined neural signatures of the stimuli. Our behavioural results suggested that masking was effective in the remaining participants and there were clear differences in the neural signatures for visible compared with invisible stimuli. Failure of masking due to eye movements is therefore unlikely.

Taken together, our present findings contribute to our understanding of unconscious processing in the early visual cortex. Without conscious awareness, the early visual cortex encodes only features and does not integrate local features into global object representations. This finding could provide an explanation for previous behavioural findings on unconscious visual processing and implies that there are fundamental limits to what information can be processed unconsciously. Our results also make testable predictions for future research: representations of visible stimuli in higher category-selective brain regions should not generalize to invisible stimuli when stimuli are partially occluded, such as when viewing faces or houses through the gaps in a fence.

## Data Availability

Stimulus presentation code and behavioural data, processed fMRI data and analysis code for reproducing the results are publicly available at: https://osf.io/u9tvp. Local regulations and ethical approvals do not permit public sharing of potentially reidentifiable brain images in native space. Provisions under *Te Tiriti o Waitangi* governing data sovereignty of Māori participants also preclude the reuse of data beyond the original purpose of the study. Additional data may be shared conditionally upon request.
